# Association of pre-pregnancy body mass index and rate of weight gain during pregnancy with maternal indicators of cardiometabolic risk

**DOI:** 10.1038/s41387-021-00178-9

**Published:** 2021-11-25

**Authors:** Luz Isabel Omaña-Guzmán, Luis Ortiz-Hernández, Mónica Ancira-Moreno, Vanesa Morales-Hernández, Marie S. O’Neill, Felipe Vadillo-Ortega

**Affiliations:** 1 Doctorado en Ciencias Biológicas y de la Salud, Universidad Autónoma Metropolitana, Mexico City, Mexico; 2 Unidad de Vinculación Científica de la Facultad de Medicina UNAM, Instituto Nacional de Medicina Genómica, Mexico City, Mexico; 3 Departamento de Atención a la Salud, Universidad Autónoma Metropolitana, Mexico City, Mexico; 4 Departamento de Salud, Universidad Iberoamericana, Mexico City, Mexico; 5 Biología de la Reproducción, Instituto Nacional de Perinatología Isidro Espinosa de los Reyes, Mexico City, Mexico; 6 Epidemiology and Environmental Health Sciences, School of Public Health, University of Michigan, Ann Arbor, MI USA

**Keywords:** Epidemiology, Risk factors

## Abstract

**Background/objective:**

Changes in metabolism and extensive hemodynamic adjustments occur during normal pregnancy. The presence of maternal obesity imposes an overload to these physiological adaptations that may result in increased risk for the development of cardiometabolic complications during and after pregnancy. The aim of this study is to describe total cholesterol (TC), triglycerides (TG), glucose, and arterial blood pressure (BP) trajectories and to analyze the association of these cardiometabolic risk indicators during pregnancy with pre-pregnancy body mass index (pBMI) and monthly gestational weight gain (MGWG).

**Subjects/methods:**

A prospective cohort study of pregnant women was conducted in Mexico City. Monthly samples of blood were taken during clinical follow-up and biochemical and blood pressure were measured during each visit. Adjusted linear mixed-effect regression models were fit to describe the trajectories of these biomarkers during pregnancy and to analyze the association with pBMI and MGWG.

**Results:**

Seven hundred and twenty women were included of which 16.6% had pre-gestational obesity, 33.2% had pre-gestational overweight, 45.8% had normal pBMI and 4.4% had pre-gestational underweight. Women with pre-gestational obesity had higher lipids concentrations in the beginning of pregnancy (TC: $\hat \beta$ = 33.08, *p* = 0.010; TG: $\hat \beta$ = 31.29, *p* = <0.001) but the concentrations increased less than in women with normal pBMI (TC: $\hat \beta$ = −14.18, *p* = 0.001; TG: $\hat \beta$ = −5.42, *p* < 0.001). By the end of pregnancy, women with pre-gestational obesity had lower concentrations of lipids than women with normal pBMI. By contrast, women with pre-gestational obesity had higher glucose concentrations and higher BP levels than women with normal pBMI over pregnancy.

**Conclusions:**

pBMI is differentially associated with longitudinal trajectories of maternal biochemical markers of cardiometabolic risk. MGWG did not significantly affect the biochemical indicators or BP trajectories. Our results suggest that pBMI is more relevant to predicting adverse cardiometabolic markers trajectories during pregnancy than MGWG.

## Introduction

Gradual changes occur in maternal metabolism and physiology during pregnancy in order to support maternal and fetal requirements [1–3]. Hyperlipidemia occurs gradually throughout normal pregnancy, characterized by increased maternal circulating cholesterol and fatty acids (FA) which are essential for fetal growth and development [4]. Additionally, maternal glucose concentrations change throughout pregnancy as a consequence of increased insulin resistance [5]. Changes also occur in the cardiovascular system to ensure an adequate uteroplacental circulation for the optimal fetal nutrient supply [6, 7].

Serum total cholesterol (TC), triglycerides (TG), and glucose levels, as well as arterial blood pressure (BP), are considered indicators of cardiometabolic risk because increased values are associated with the development of cardiovascular and metabolic diseases [8]. Abnormal values of these markers during pregnancy have been associated with the development of maternal complications and adverse perinatal outcomes such as preeclampsia [9], gestational diabetes mellitus [10, 11], preterm delivery [9, 12], large for gestational age babies [9, 13], low birth weight and atherosclerosis in the fetus [14]. Alterations in BP trajectories are related to adverse outcomes for the mother and offspring, such as pregnancy-induced hypertension, preeclampsia, preterm birth, intrauterine growth restriction, and increased blood pressure in offspring [6, 7, 15, 16].

Scarce evidence exists for how common conditions such as obesity may affect maternal physiology, modifying cardiometabolic risks during and after pregnancy. The pre-pregnancy body mass index (pBMI) and gestational weight gain (GWG) are two variables that have been used as indicators of nutritional status and adiposity during pregnancy. However, weight gain patterns during pregnancy are strongly linked to perinatal outcomes, lactation performance, postpartum weight retention, and cardiovascular and other chronic diseases. Pre-pregnancy BMI has been shown to be an independent predictor of adverse effects on mother and offspring [17]. For this reason, an effort to evaluate these indicators in different populations may lead to better identification of women at risk during pregnancy.

The effect of pBMI on lipid trajectories in pregnancy has been previously evaluated [18–22], showing that they are different in pregnant women with obesity. Less is known about the effect of GWG on these biomarkers. No studies were identified analyzing the association of pBMI and monthly gestational weight gain (MGWG) on glucose concentrations throughout pregnancy. However, evidence that obesity during pregnancy constitutes a higher risk for the development of gestational diabetes is available [23, 24]. Regarding BP, studies have found a positive association of pBMI with systolic, diastolic, and mean BP (SBP, DBP, and MAP) [25–27]. Likewise, in one study [28], GWG was positively associated with SBP and DBP.

Most studies that have assessed the trajectories of lipids and BP during pregnancy, as well as their association with pBMI or MGWG, have been performed in high-income countries [16, 19–21, 28, 29]. Except for Brazil [21], studies are lacking from Latin American countries with a high prevalence of obesity, in which the combination of the genomic background and environmental determinants may present additional risk factors for pregnancy complications and later in life increased risk for cardiovascular diseases [30–32].

The aim of this study is to describe, during pregnancy, the trajectories of TC, TG, glucose, and BP and to analyze the association of these indicators of cardiometabolic risk with pBMI and MGWG in Mexican pregnant women.

## Subjects and methods

### Design and population

The Pregnancy Research on Inflammation, Nutrition & City Environment: Systematic Analyses (PRINCESA) prospective cohort study was conducted at the *Hospital Materno Infantil Inguarán* in Mexico City. The PRINCESA cohort was established to evaluate the effect of diverse environmental exposures during pregnancy on fetal development and maternal health [32, 33]. Participants were recruited from 2010 to 2015, they entered follow-up no later than 18 weeks of gestation and were followed monthly (*n* = 794). Participants received prenatal medical examinations and fetal growth and development assessment, and provided data on nutritional status, self-reported daily physical activity, and exposure to environmental pollutants. At each hospital visit, biological samples were collected for clinical follow-up and research objectives. The inclusion criteria for the present analysis were: pregnant women who had at least two measurements of anthropometric and biochemical data and were between the ages of 18 and 49 years. Women who developed any pregnancy complications including gestational diabetes and preeclampsia were excluded as well as women reporting active smoking. The study was approved by the Ethics Committee of the National Institute of Genomic Medicine, the National Autonomous University of Mexico and the Health Ministry of Mexico City, and the Institutional Review Board of the University of Michigan. All participants signed an informed consent letter.

### MGWG and pBMI

The pBMI was calculated using the height and weight measured in the first visit (less than 18 weeks of gestation). We decided to use this measurement because we have previously identified that a systematic error in pBMI was present in this population and for the great majority of participants, BMI was calculated before the linear phase of weight gain during pregnancy [34]. Weight and height measurements were made using the Lohman technique [35] by personnel whose training was standardized by the Habitch method [36]. The height was measured using a Seca stadiometer with a precision of 0.1 cm. At each follow-up visit, the participant’s weight was measured with a Tanita scale with a precision of 0.01 kg, and the weight rounded to the nearest 100 grams. Considering the design of our study, which was based on the monthly structure of prenatal care, we used GWG as MGWG, and this was calculated as the difference in weight between two consecutive months of the pregnancy. Total gestational weight gain was calculated as the difference between the first measured weight at the beginning of follow-up and the last measured weight at week 36 of gestation and was categorized according to IOM recommendations for clinical use.

### Cardiometabolic risk indicators

Serum concentrations of TC, TG, glucose, SBP, DBP, and MAP were considered as indicators of cardiometabolic risk. At each follow-up visit, a venous blood sample was taken after 8 h of fasting using sodium fluoride/potassium oxalate Vacutainer tubes to inhibit glucose metabolism (BD Vacutainer, Mexico). Serum was separated within 3 h after sampling. Samples were processed using the Adaltis automated system and SpinReact reagents (Spin React, Clinical Diagnostics, Paris, France). BP was measured with a mercury sphygmomanometer according to the American Heart Association procedure [37]. TC was determined by the method of enzymatic hydrolysis and oxidation and TG concentrations were measured after hydrolysis in the automatic analyzer. For the statistical analyses, three or four standard deviations (SD) were used to identify and eliminate outliers. For TC and glucose, ±3 SD were used to identify outliers; the cut-off values corresponded to TC values lower than 67 mg/dL and greater than 397 mg/dL, and glucose values of 50 and 122 mg/dL, respectively. For TG, values higher than 500 mg/dL (4 SD above the mean) were eliminated; the lowest value of TG was 23 mg/dL, which is biologically plausible. These elimination criteria coincide with the ranges of lipids [18, 19, 21] and glucose [38] that have been found it in other populations of pregnant women.

### Covariates

Data about age, education, marital status, and parity of the participants were obtained from the questionnaires that were applied in the first visit. The gestational age at the first visit was calculated using the date of the last menstruation and confirmed with gestational ultrasound evaluation occurring at less than 14 weeks. The weeks of pregnancy were categorized into months according to the following classification: month 2, weeks 5 to 8.6; month 3, weeks 9 to 13.6; month 4, weeks 14 to 17.6; month 5, weeks 18 to 22.6; month 6, weeks 23 to 27.6; month 7, weeks 28 to 31.6; month 8, weeks 32 to 35.6; month 9, weeks 36 to 40.6. Dietary information was collected by a 24 h recall multiple-pass method.

To assess physical activity, a self-applied questionary of daily activities, included weekdays and weekends was filled by all participants at least in three different moments during gestation.

Clinical information of delivery outcomes for all participants and their babies directly from the clinical records was retrieved.

### Statistical analysis

Descriptive analyses were performed to characterize the study population. For continuous variables, means and SD were calculated. For categorical variables, absolute and relative frequencies were estimated. To describe TC, TG, glucose, and BP trajectories and to assess their associations with pBMI and MGWG, mixed models with intercepts and random slopes were estimated [39]. This type of model is used when the observations do not fulfill the premise of independence of observations, which is required when ordinary least squares regression models are used.

First, to verify which pattern (linear, quadratic, or cubic) best characterized the trajectories of the cardiometabolic risk indicators and thus choose an appropriate model, the observed values of TC, TG, glucose, SBP, DBP, and MAP were plotted against those predicted by the different regression mixed models with linear, quadratic and cubic functions of pregnancy months. To choose the best model for each dependent variable, the log-likelihood of the models was compared. Then, to assess the association of cardiometabolic risk indicators with pBMI, and MGWG crude and adjusted mixed models were estimated. Furthermore, in order to distinguish the cross-sectional and longitudinal effects of pBMI, an interaction between this variable and the month of pregnancy was tested in the models. Theoretically, MGWG is associated with pBMI, i.e., women with obesity tend to have higher GWG than those with normal weight. Therefore, it was necessary to assess whether these two variables have independent effects on maternal cardiometabolic risk. For this reason, the mixed-effects models were performed with and without the MGWG variable for each cardiometabolic risk indicator. The models were adjusted by the following covariates: age, education, marital status, parity, and intake of energy and micronutrients. No difference in physical activity among participants was found hence, it was not included as a covariable. To better visualize the results, TC, TG, glucose, SBP, DBP, and MAP trajectories for pBMI subgroups were plotted utilizing the final adjusted mixed models. For these plots, fixed values were used for the covariates. For interactions, a statistically significant value was considered when p-value <0.1, while for the other analysis a *p*-value <0.05 was considered as significant. The analyses were performed in STATA/SE 15.0.

## Results

### Baseline characteristics

Seven hundred and twenty women were included in this study (Supplemental Fig. 1). The median gestational age at enrollment was 12.6 weeks (range 8.1–18). The number of visits during clinical follow-up ranged from 2 to 8 with an average of 4. To assess that there would be no differences regarding the number of visits between the different pBMI groups, a chi-square test was performed. No differences were found between these two variables (*p* = 0.91). The baseline characteristics of participants are shown in Table 1. Descriptive data about MGWG, lipids, glucose, and BP are shown in Supplemental Table 1. Total gestational weight gain was not retrieved at a term of gestation in all women because Mexico City’ Health System randomly referred women for delivery to eight different hospitals and we missed final weight in 50% of women. The total gestational weight gain average was only 8.8 ± 5.6 kg, making 52.3% of the population insufficient GWG according to IOM recommendation.

**Table 1:** Baseline characteristics of participants from the PRINCESA cohort, Mexico City.

	Mean ± SD
Age (years)	25 ± 5.9
Height (cm)	156 ± 6.0
Pre-gestational weight (kg)	62.5 ±13.6
Pre-gestational BMI (kg/m^2^)	25.6 ± 5.2
Total gestational weight gain (kg)	8.8 ± 5.6
Pre-gestational BMI	% (*n*)
Low weight	4.4 (32)
Normal	45.8 (330)
Overweight	33.2 (238)
Obesity	16.6 (120)
Education	
Elementary/no studies	11.3 (81)
Secondary	45.2 (324)
High school/technical	35.6 (255)
Bachelors degree	7.8 (56)
Marital status	
Married	21.5 (154)
Single/divorced	26.3 (189)
Consensual union	52.2 (374)
Parity	
1	488 (66.7)
2	222 (30.4)
3+	21 (2.9)

*BMI Body Mass Index, SD* standard deviation

Macronutrients and energy intake were added to models as covariates but did not have a significant association with the cardiometabolic risk indicators, and their inclusion did not modify the regression coefficients of the pBMI or MGWG. However, the inclusion of these intakes decreased the number of observations in the models. Therefore, we decided not to include them in the final models.

To identify the best fit for cardiometabolic risk indicators trajectories during pregnancy and to choose an appropriate model for analysis, we plotted the observed values against predicted values using either linear, quadratic, and cubic functions of time. The goodness of fit of the model as measured by log-likelihood for TC levels according to pregnancy progression was best when a quadratic function was used. Changes in TG concentrations by pregnancy month were fit best by a linear function. Glucose concentration values according to pregnancy month were best modeled using a cubic term of time. Changes in SBP, DBP, and MAP during pregnancy progression were best characterized using a cubic term of time in the model, although the differences among models were minimal.

### Association between cardiometabolic risk trajectories with MGWC and pBMI

**Fig. 1:**
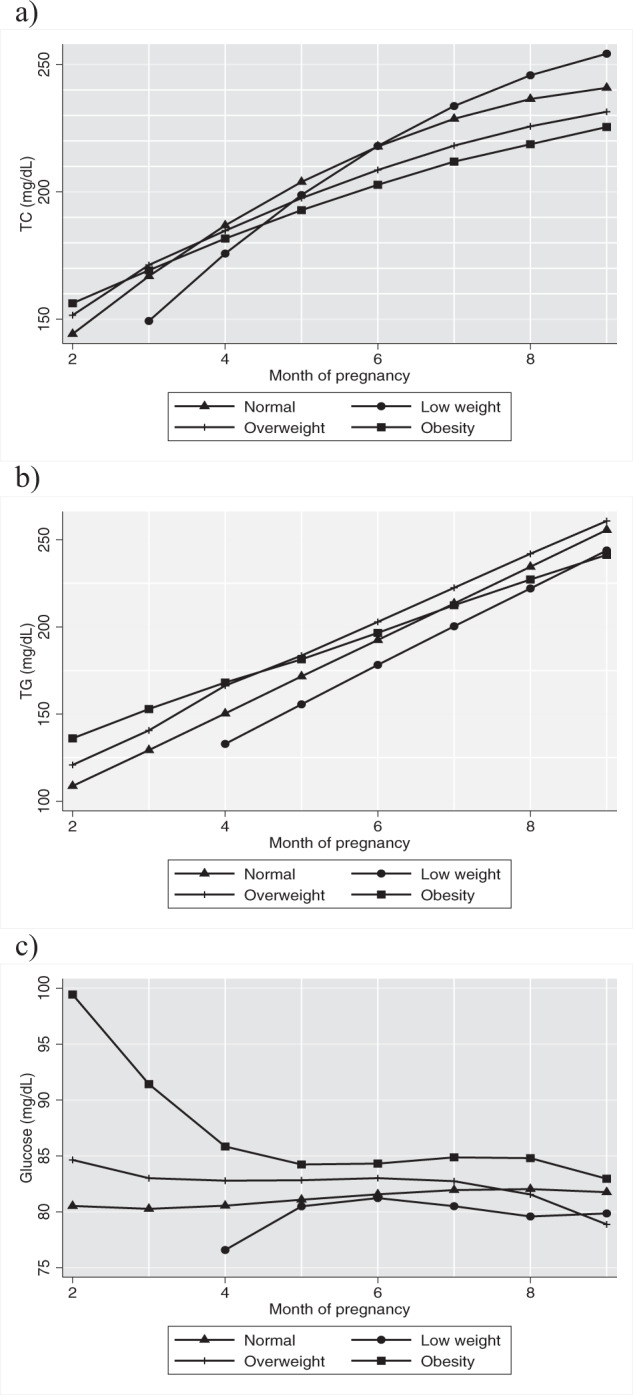
Adjusted TC, TG, and glucose trajectories through pregnancy by pBMI groups. **a** Adjusted TC trajectories by pBMI groups. **b** Adjusted TG trajectories by pBMI groups. **c** Adjusted glucose trajectories by pBMI groups. pBMI pre-pregnancy body mass index, TC total cholesterol, TG triglycerides. Adjusted models controlled for: age, height, education, marital status, month of pregnancy, parity.

The mixed regression models introducing cardiometabolic risk indicators as outcomes and pBMI and MGWG during pregnancy as exposures are shown in Tables 2 and 3. Women with overweight and pre-gestational obesity had higher TC concentrations at the beginning of pregnancy compared to those with normal pBMI ($\hat \beta$ = 26.62; $\hat \beta$ = 33.08, coefficients for overweight and obesity, respectively). In addition, as the pregnancy progressed, the increase of TC among overweight and obese women was lower ($\hat \beta$ = −10.35, $\hat \beta$ = −14.18 for interaction with month, respectively) than in women with normal pBMI, and as time progressed the differences between these increases diminished ($\hat \beta$ = 0.69, $\hat \beta$ = 0.93 for interaction with month^2^, coefficients for overweight and pre-gestational obesity, respectively). Thus, women with pre-gestational obesity had lower TC concentrations than women with normal pBMI at the end of pregnancy, as can be seen in the adjusted trajectories plotted (Fig. 1a). When MGWG was added to the model, the pBMI coefficients lost statistical significance (see models 1 and 2 for TC in Table 2). Therefore, it was not included in the model that was used to plot.

**Table 2:** Adjusted mixed model results for biochemical risk factors.

	TC model 1^a^		TC model 2^a^		TG		Glucose	
	*n* = 718		*n* = 714		*n* = 714		*n* = 713	
	$\hat \beta$	*p*	$\hat \beta$	*p*	$\hat \beta$	*p*	$\hat \beta$	*p*
Month of pregnancy	31.25	<0.001	26.69	<0.001	21.03	<0.001	−2.57	0.561
Month of pregnancy^b^	−1.57	<0.001	−1.27	<0.001			0.55	0.465
Month of pregnancy^c^							−0.03	0.427
pBMI								
Low weight	−36.92	0.135	13.08	0.749	−18.77	0.297	−68.37	0.370
Overweight	26.62	0.008	12.83	0.404	17.00	0.024	9.25	0.465
Obesity	33.08	0.01	4.27	0.836	31.29	0.001	45.73	0.011
*Interaction*								
pBMI × month								
Low weight	8.03	0.336	−7.18	0.638	1.4	0.612	31.7	0.387
Overweight	−10.35	0.002	−5.78	0.232	−1.43	0.187	−4.2	0.532
Obesity	−14.18	0.001	−5.50	0.396	−5.42	<0.001	−19.92	0.033
*Interaction*								
pBMI × month^b^								
Low weight	−0.24	0.722	0.88	0.442			−4.77	0.428
Overweight	0.69	0.014	0.34	0.362			0.80	0.489
Obesity	0.93	0.009	0.34	0.498			3.03	0.056
*Interaction*								
pBMI*month^c^								
Low weight							0.23	0.428
Overweight							−0.05	0.396
Obesity							−0.15	0.079
MGWG			0.18	0.683	0.06	0.931	0.16	0.352

Adjusted models controlled by: pBMI, MGWG, age, height, education, marital status, month of pregnancy, and parity.

*MGWG* monthly gestational weight gain, *pBMI* pre-pregnancy body mass index, *TC* total cholesterol, *TG* triglycerides.

^a^Model 1 controlled by: pBMI, age, height, education, marital status, month of pregnancy and parity; Model 2 controlled by: pBMI, age, height, education, marital status, month of pregnancy and parity + MGWG.

^b^Quadratic function of month of pregnancy.

^c^Cubic function of month of pregnancy.

**Table 3:** Adjusted mixed models results for blood pressure.

	SBP		DBP		MAP	
	*n* = 710		*n* = 708		*n* = 706	
	$\hat \beta$	*p*	$\hat \beta$	*p*	$\hat \beta$	*p*
Month of pregnancy	0.64	0.851	5.03	0.058	3.84	0.145
Month of pregnancy^a^	−0.31	0.588	−0.98	0.031	−0.83	0.063
Month of pregnancy^b^	0.03	0.346	0.06	0.011	0.06	0.019
pBMI						
Low weight	−72.87	0.194	4.51	0.922	−23.36	0.595
Overweight	7.80	0.411	22.60	0.003	18.19	0.014
Obesity	15.26	0.251	31.61	0.003	27.12	0.008
*Interaction*						
pBMI × month						
Low weight	35.68	0.191	−1.29	0.953	12.10	0.568
Overweight	−2.19	0.661	−10.21	0.011	−8.10	0.037
Obesity	−4.60	0.507	−14.69	0.007	−12.25	0.020
*Interaction*						
pBMI × month^a^						
Low weight	−5.24	0.181	−0.01	0.997	−2.09	0.529
Overweight	0.24	0.777	1.58	0.021	1.25	0.059
Obesity	0.70	0.546	2.44	0.008	2.06	0.020
*Interaction*						
pBMI*month^b^						
Low weight	0.29	0.179	0.01	0.972	0.11	0.511
Overweight	−0.01	0.900	−0.08	0.037	−0.06	0.093
Obesity	−0.04	0.586	−0.13	0.008	−0.11	0.019
MGWG	0.17	0.168	−0.02	0.805	0.03	0.746

Adjusted models controlled by: pBMI, MGWG, age, height, education, marital status, month of pregnancy and parity.

*MGWG* monthly gestational weight gain, *pBMI* pre-pregnancy body mass index, DBP dyastolic blood presssure, SBP systolic blood pressure, MAP mean arterial pressure

^a^Quadratic function of month of pregnancy.

^b^Cubic function of month of pregnancy.

Regarding TG concentrations, these were higher in women with pre-gestational overweight or obesity than in women with normal pBMI ($\hat \beta$ = 17.00, $\hat \beta$ = 31.29, respectively) but these parameters had smaller increases throughout the pregnancy in the case of women with obesity ($\hat \beta$ = −5.42 for interaction with month). Women with pre-gestational obesity ended the pregnancy with lower TG concentrations than women with normal pBMI (Fig. 1b).

Similar to TG, glucose concentrations were higher in women with pre-gestational obesity ($\hat \beta$ = 45.73). However, glucose concentrations in those women decreased over pregnancy ($\hat \beta$ = −19.92 for interaction with month) whereas in women with normal pBMI increased. As time progressed the differences between these trajectories diminished and at the end of pregnancy a slightly decrease was observed in glucose concentrations in women with pre-gestational obesity ($\hat \beta$ = 3.03, $\hat \beta$ = −0.15 for interaction with month^2^ and month^3^, respectively). At the end of pregnancy, women with obesity had slightly higher glucose concentrations than women with an adequate pBMI (Fig. 1c). After adjusting for MGWG, the association of pBMI and glucose did not change.

The MGWG was not associated with TG ($\hat \beta$ = 0.06, p = 0.931) and glucose concentrations ($\hat \beta$ = 0.16, *p* = 0.352).

**Fig. 2:**
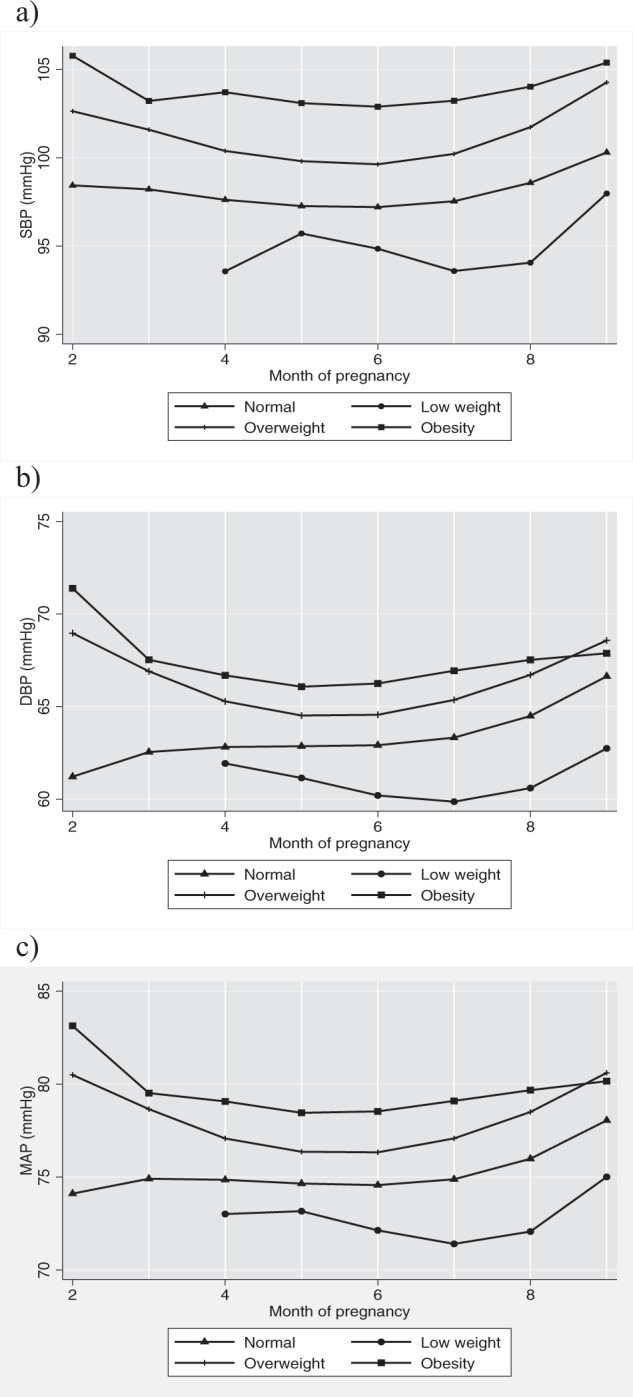
Adjusted SBP, DBP, and MAP trajectories through pregnancy by pBMI groups. **a** Adjusted SBP trajectories by pBMI groups. **b** Adjusted DBP trajectories by pBMI groups. **c** Adjusted MAP trajectories by pBMI groups. pBMI pre-pregnancy body mass index, SBP systolic blood pressure, DBP diastolic blood pressure. Adjusted models controlled for: age, height, education, marital status, month of pregnancy parity and MGWG.

The pBMI had no significant effect on SBP throughout pregnancy since the interaction between pBMI and month of pregnancy was not significant (Fig. 2a). MGWG was not associated with SBP levels during pregnancy.

DBP and MAP had similar tendencies: at the beginning of the pregnancy, women with pre-gestational overweight or obesity had higher levels (DBP: $\hat \beta$ = 22.60, $\hat \beta$ = 31.61; MAP: $\hat \beta$ = 18.19, $\hat \beta$ = 27.12, respectively) than those with normal pBMI. The interaction term between the month of pregnancy and pBMI was significant: women with pre-gestational overweight and obesity had decreased in DBP and MAP levels at the beginning of pregnancy (DBP: $\hat \beta$ = −10.21, $\hat \beta$ = −14.69 for interaction with month, coefficients for overweight and pre-gestational obesity; MAP: $\hat \beta$ = −8.10, $\hat \beta$ = −12.25 for interaction with month, coefficients for overweight and pre-gestational obesity, respectively). As pregnancy progressed, an increase was observed (DBP: $\hat \beta$ = 1.58, $\hat \beta$ = 2.44 for interaction with month^2^, coefficients for overweight and pre-gestational obesity, respectively; MAP: $\hat \beta$ = 1.25, $\hat \beta$ = 2.06 for interaction with month^2^, coefficients for overweight and pre-gestational obesity, respectively). At the end of pregnancy, a slightly increase was observed (DBP: $\hat \beta$ = −0.08, $\hat \beta$ = −0.13 for interaction with month^3^, coefficients for overweight and pre-gestational obesity, respectively; MAP: $\hat \beta$ = −0.06, $\hat \beta$ = −0.11 for interaction with month^3^, coefficients for overweight and pre-gestational obesity, respectively). Despite this pattern, women with pre-gestational overweight or obesity had higher DBP and MAP levels throughout pregnancy (Fig. 2b, c). MGWG was not associated with either the DBP or MAP.

## Discussion

The growing prevalence of women who are obese or overweight entering pregnancy has raised concerns to the health systems worldwide, since many pregnancy complications are associated with these conditions [40, 41]. Additional deleterious effects on the future of women’s health and child development justify a special effort directed toward understanding and controlling the mechanisms of damage of increased adiposity. In this paper, we explored the association of two indicators of adiposity in pregnant women with longitudinal changes of cardiometabolic biomarkers during normal pregnancy. We included in this study women in a range of pBMI and MGWG that ended pregnancy without pathologic metabolic or cardiovascular conditions.

We found that pBMI and MGWG are differentially associated with cardiometabolic risk indicators in a cohort of Mexican pregnant women. In general terms, pBMI, an indicator of maternal health and nutrition history, is associated with differential concentrations in lipids, glucose, and blood pressure during pregnancy. Some of our results are consistent with previous evidence associating pBMI and MGWG with lipid trajectories during pregnancy [18–21]. Women with pre-gestational overweight or obesity showed lower TC concentrations during pregnancy than normal-weight women; this is consistent with previous reports in women from Brazil [18], the United States [19], and Austria [21]. Similar results in TG concentrations were found in a previous study, characterized by lower values of TG during the second and third trimester in women with pre-gestational obesity [21]. Other studies did not show these differences [18–20], but the effect of potential confounders was not considered. As expected, women with pre-gestational obesity had higher glucose levels during pregnancy than women with normal pBMI. It is well known that maternal obesity is associated with increased insulin resistance and a higher risk for gestational diabetes [42].

Decreased circulating TC and TG and elevated glucose levels in women with overweight and obesity seem contradictory if insulin action were mediating this effect. This paradox may indicate that adipose tissue in women with pre-gestational obesity is responding better to insulin action or that the use of FA is increased in these women, involving the transport of FA and TC by the placenta [43, 44]. We know that FA cross the placenta following concentration gradient [43] and it has already been demonstrated that the concentration of TG in placental tissue and the expression of genes related to FA transporters in the placenta was higher in women with pre-gestational obesity than in women with normal pBMI [45]. Consequently, is possible that a lower increase in blood lipids in women with obesity, may be linked to the placental transfer of lipids to the fetal circulation, exposing the fetus to the abnormal concentration of FA and may be, inducing fetal adipogenesis. Further analysis of metabolomic adjustments in women with obesity may clarify if other metabolic pathways such as gluconeogenesis are activated in women with different pBMI and contribute to different glucose trajectories. Also, characteristics of fetal adipose tissue, neonatal body weight composition, and adipose tissue accretion later in children life must be explored.

Consistent with our results, higher pBMIs were associated with higher SBP, DBP, and MAP in women from other populations [46–49]. MAP always remained higher and strongly associated with pBMI in our cohort, similar to results reported previously [50]. The physiological mechanisms behind the association between pBMI and higher BP levels are thought to be the same mechanisms by which excess weight increases BP levels in a non-pregnancy condition [47]. It is known that women with hypertensive disorders during pregnancy have a greater long-term cardiovascular risk [51, 52]; however, there is also evidence that maternal obesity is associated with higher BP levels, as well as with microvascular endothelial dysfunction in normotensive pregnancies [53]. Taking into account the above, it is possible that the broad metabolic and physiological changes that occur during pregnancy in a state of maternal obesity cause alterations that increase the cardiometabolic risk in the medium and long term.

Cardiovascular disease is the actual leading cause of mortality for women around the globe and conditions such as pregnancy complicated with preexistent obesity may be relevant contributors to this health problem [54]. Therefore, observations in this study allow to set a starting point to the follow-up of these women in the future and to evaluate their cardiovascular and metabolic health in the medium and long term, correlating with conditions during pregnancy.

We concluded that pBMI is a stronger modifier of cardiometabolic risk indicators rather than MGWG since this variable was not associated with maternal risk indicators. Considering the design of our study, in which participants were evaluated on a monthly basis, we decided that it was better to evaluate GWG as the change in weight from month to month (MGWG) than the total GWG. In this way, we had more detailed information from the GWG. It has been shown that excessive GWG is associated primarily with an increase in adipose tissue and not with lean mass [55] and an excessive accretion of adipose tissue during pregnancy could lead to metabolic dysfunction similar to non-pregnant conditions. However, in our study, no association between MGWG and cardiometabolic indicators was found. Considering that the GWG is related to pBMI, we tested whether the relationship of cardiometabolic risk with pBMI was independent of MGWG. When the MGWG was introduced in the TC model, the significance of the association between it and pgBMI was lost, which suggests that MGWG could be a mediating or confounding variable. Since the variability of MGWG depends on pBMI is possible that women with obesity gain more weight, which can promote higher hepatic production of TC [56]. Another possibility is that both variables have independent effects on TC. For the other cardiometabolic indicators, pBMI is an independent predictor of cardiometabolic risk trajectories.

An advantage of this study is its longitudinal design, which allowed us to model the trajectories (i.e., monthly changes) of TC, TG, SBP, DBP, and MAP throughout the pregnancy, something that had not been previously described in Latin American women. One novel aspect of our study was the evaluation of the association between pBMI and glucose trajectories during pregnancy.

The monthly measurements allowed us to obtain more detailed information about the changes in these indicators of cardiometabolic risk during pregnancy and therefore to choose adequate modeling of time to describe the association of these biomarkers with pBMI and MGWG. Our models permitted the modeling the non-linear trajectories of TC, glucose, SBP, DBP, and MAP and appropriately addressed the within-subject dependence of measures. Another advantage of our study is that we estimated the trajectories according to pBMI adjusting for potential confounding variables, which allowed a better approach to isolate the effect of pBMI and MGWG on cardiometabolic risk indicators.

One limitation of this study is that not all women have the eight repeated measurements that were considered in the analysis. However, the mixed effect allowed us to consider all the available information because it considers all data of women who had at least one measurement. Another limitation is that our results cannot be extrapolated to other populations because we did not have a probabilistic sample. The institution where the study was conducted belongs to the Ministry of Health of Mexico City and gives care to the population that is not affiliated with the major social security system. In Mexico City, low-income women are covered by the local government health system [57].

In conclusion, our results show that pBMI is an important predictor of biochemical and BP modifications during pregnancy, which may increase the cardiometabolic risk during the actual pregnancy and later in women’s life. We suggest further follow-up of this group of women, to evaluate and prevent cardiometabolic complications later in life, as well as to correlate the different risks factor trajectories with effects on fetal growth and development. Our results support the need for additional efforts directed towards promoting an adequate pBMI and evaluating new clinical stratification of pregnant women based on cardiometabolic risk.

## Supplementary information

Supplemental Figure 1

Supplemental Table 1
