# Supplementary figures and images for: Association of pre-pregnancy body mass index and rate of weight gain during pregnancy with maternal indicators of cardiometabolic risk
Source: Nutr Diabetes. 2021 Nov 25;11:36. doi: 10.1038/s41387-021-00178-9 (PMC8616911; doi:10.1038/s41387-021-00178-9)

Supplemental Figure 1. Study Flow

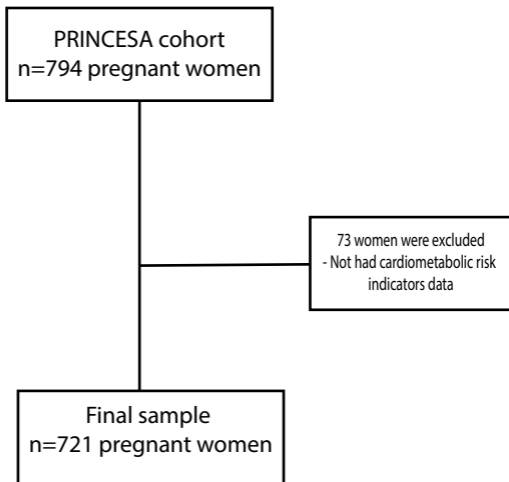

Supplement: Supplementary file 1 — Supplemental Figure 1 [file 41387_2021_178_MOESM1_ESM.pdf]
